# Daptomycin-Tobramycin Cement Beads have Lethal Local Antibacterial Effect in Resistant Periprosthetic Joint Infections

**DOI:** 10.7759/cureus.5726

**Published:** 2019-09-22

**Authors:** Vivek Jagadale, Robert Achilike, Keith M Nord

**Affiliations:** 1 Orthopedics, University of Arkansas for Medical Sciences, Little Rock, USA

**Keywords:** daptomycin, tobramycin, pmma beads, bone cement, prosthetic joint infection, total knee arthroplasty, total hip arthroplasty, staph aureus

## Abstract

Background

Resistant periprosthetic joint infection (PJI) can be a devastating complication of surgery and is difficult to treat. We attempted to identify the utility of Daptomycin/Tobramycin-impregnated polymethylmethacrylate (PMMA) beads in resistant PJIs.

Methods

Charts of patients with hip or knee PJI at a single academic medical center, with surgeries performed from May 2008 through May 2018, were reviewed retrospectively. The diagnosis of PJI was consistent with the Musculoskeletal Infection Society recommendations. Prosthetic joints underwent radical anterior-posterior synovectomy and placement of antibiotic cement beads in the infected joint. Clinical cure rate and local intraarticular antibiotic concentration were measured.

Results

Forty-four patients experienced 53 episodes of PJI, requiring 88 surgeries. There was a fairly even split between hip and knee PJI. The cure rate was 92% (11/12) for patients who had any infection with methicillin-resistant staphylococci during the evaluation period, compared with 62% (13/21) for patients with methicillin-susceptible *Staphylococci*. On days one and seven, the addition of tobramycin increased daptomycin concentrations by 47% and 3374%, respectively, for beads made onsite compared to elution of daptomycin alone. Elution increased by 44% and 8394%, respectively, for commercial beads compared to beads fabricated onsite.

Conclusions

Daptomycin-Tobramycin-loaded PMMA beads are safe and powerful bactericidal agents in prosthetic joint infections.

## Introduction

Periprosthetic joint infection (PJI) can be a devastating complication of surgery and is difficult to treat, often resulting in repeated surgeries, prolonged management, significant costs, disability, and death [1]. The Musculoskeletal Infection Society (MSIS) has recently established guidelines for the diagnosis of PJI of the hip and knee [2]. Treatment usually requires a combination of surgical and medical management [3-4]. Surgery may range from simple debridement to a two-stage exchange of the prosthetic device to amputation, among other procedures [4] retention of the original components may be appropriate in some circumstances [5]. Antibiotic-impregnated polymethylmethacrylate (PMMA) beads or other devices often have been implanted for a local effect and have been associated with improved outcomes [4,6].

Vancomycin has been the drug of choice for treating methicillin-resistant strains of *Staphylococcus aureus* (MRSA) and coagulase-negative *Staphylococci*, the most common causes of PJI [3-4]. Because vancomycin only has activity against gram-positive organisms, it is usually combined with an aminoglycoside. In the United States, tobramycin is generally used instead of gentamicin in implanted devices because the latter is not commercially available in powder form. The addition of an aminoglycoside offers several possible advantages. The combination of an aminoglycoside with a cell wall active agent has synergistic activity against gram-positive organisms, although the clinical relevance of this is unclear [7-8]. An aminoglycoside theoretically may decrease the emergence of resistance to other agents [9]. Finally, an aminoglycoside may enhance the elution of other antibiotics through increased porosity of bone cement, although this has not been published previously for Daptomycin [7,9-11].

However, vancomycin has several limitations, mainly decreased susceptibility of *S. aureus* [7,9,11-14]. This is reflected by increasing minimum inhibitory concentrations (MICs) at some institutions, known as “MIC creep” [15-16]. Reduced efficacy of vancomycin against *S. aureus* with MICs ≥1 μg/mL have been shown by Lodise and others, even though MIC of ≤2 μg/mL is defined as susceptible by the Clinical and Laboratory Standards Institute, increased doses have been used to combat this, but worse outcomes have been associated with vancomycin MICs ≥2 μg/mL, regardless of whether or not troughs ≥15 μg/mL were achieved, and other outcomes have also been shown to be suboptimal [11-13].

Daptomycin is a cyclic lipopeptide antibiotic active against a wide range of gram-positive pathogens [17]. Some vancomycin-intermediate *S. aureus* (VISA) and heteroresistant VISA (hVISA) also exhibit daptomycin nonsusceptibility [18]. The exact mechanisms of daptomycin nonsusceptibility are likely multifactorial, the frequency of daptomycin nonsusceptibility is unknown but remains low, including for *S. aureus* strains with MICs of 2 μg/mL [17-19]. However, the clinical significance of daptomycin nonsusceptibility is unclear when local therapy is used. Although not indicated for PJI, intravenous (IV) daptomycin appears safe and effective for the treatment of bone and joint infections [6], and daptomycin is increasingly utilized as an alternative to vancomycin for musculoskeletal infections in situations in which the latter drug has failed or cannot be used.

 Despite the evident utility of intravenous (IV) daptomycin for bone and joint infections, clinical outcomes of local daptomycin beads for PJI have not been reported yet. The primary objective of this study was to determine the effectiveness in humans of daptomycin/tobramycin-loaded PMMA beads in PJI, with a secondary objective of measuring daptomycin and tobramycin elution from those beads.

## Materials and methods

Charts of consecutive patients who experienced hip or knee PJI at a single academic medical center, with surgeries performed from May 2008 through May 2018, were reviewed retrospectively. A standard approach was used for the diagnosis of PJI, consistent with the Musculoskeletal Infection Society recommendations [2]. The study received expedited Institutional Review Board approval and was conducted in accordance with the Declaration of Helsinki.

Regardless of the surgical procedure, meticulous debridement was performed in all patients. Prosthetic joints that were almost one month old underwent fixed component retention with radical anterior-posterior synovectomy and placement of three antibiotic-PMMA chains in the suprapatellar pouch. Prosthetic joints that were of age greater than one month or age indeterminate were replaced using a modified two-stage procedure involving articulating antibiotic-PMMA spacers plus intramedullary bead implantation, as described previously, second-stage revision or intra-articular bead removal took place an average of 12 weeks after the first surgery, with timing based on inflammatory markers (i.e., erythrocyte sedimentation rate, C‑reactive protein) and drainage [20]. Aerobic and anaerobic cultures were obtained at the time of incision or within 24 hours pre-operatively. Clinical cure was defined as negative inflammatory markers or the absence of signs and symptoms of PJI at the previous site of infection after an antimicrobial-free period of six to 12 weeks.

Antibiotic-PMMA beads consisted of 4 g of daptomycin powder (Cubist Pharmaceuticals, Lexington, MA) and 4.8 g of tobramycin powder (X-Gen Pharmaceuticals, North Port, NY) mixed with 40 grams of Palacos® Bone Cement (Biomet, Warsaw, IN; i.e., a final concentration of 8.2% and 9.8%for daptomycin and tobramycin, respectively). Antibiotics and PMMA were mixed as described previously and were either obtained commercially (New England Compounding Center, Framingham, MA) or fabricated onsite, using 25 beads per chain and a diameter of 6.4 to 7-mm per bead strung on 20-gauge surgical wire [20]. Systemic antimicrobials were administered as appropriate, with empiric therapy of daptomycin 6 mg/kg followed by culture-directed treatment. Systemic antibiotics were continued for six weeks, with fluconazole continued for a longer duration when indicated. Data were analyzed using mean, median, and range values.

## Results

Forty-four patients experienced 53 episodes of PJI, requiring 88 surgeries. Demographic information is shown in Table 1. 

**Table 1:** Demographic and clinical information

Age (year), median (range)	71 (30, 89)
Gender, n/N (%)	
Female	20/44 (46)
Male	24/44 (55)
Race, n/N (%))	
Black	7/44 (16)
White	37/44 (84)
Location, n/N (%)	
Hip	37/88 (42)
Knee	49/88 (56)
Both hip and knee	2/88 (2)
Classification at surgery, n/N (%)	
Acute	32/88 (36)
Chronic	46/88 (52)
Acute on chronic	1/88 (1)
Superficial infection only	1/88 (1)
No active infection	8/88 (9)

The study consisted mostly of elderly, white patients. There was a fairly even split between hip and knee PJI. Most episodes were categorized as chronic PJI during surgery, but the acute infection was also common. Patients classified as having no active infection were seen during their last surgery, other than one patient who was noted to have a superficial infection for which daptomycin-tobramycin-PMMA beads were implanted. Operative cultures are shown in Table 2. 

**Table 2:** Cultures obtained operatively during 88 surgeries MRSA, methicillin-resistant Staphylococcus aureus; MRSE, methicillin-resistant *Staphylococcus epidermidis*; MSSA, methicillin-susceptible *S. aureus*; MSSE, methicillin-susceptible S*. epidermidis*; NOS, not otherwise specified ^a^Includes two patients with vancomycin-resistant Enterococci ^b^Group B Streptococci (3); group C streptococci, *S. pneumoniae*, *S. gordonii*, β-hemolytic *Streptococci* NOS ^c^Gram-positive cocci NOS (2); Staphylococcus hominis, Corynebacterium sp. ^d^*Achromobacter* sp., *Citrobacter diversus*, *Serratia marcescens*

Organisms	n (%)
Gram-positive organisms	
MSSA	7 (8)
MRSA	12 (14)
MSSE	5 (6)
MRSE	12 (14)
Enterococci^a^	7 (8)
Streptococci^b^	7 (8)
Other^c^	4 (5)
Gram-negative organisms	
Escherichia coli	4 (5)
Proteus mirabilis	2 (2)
Pseudomonas spp.	3 (3)
Other^d^	3 (3)
Mixed flora	7 (8)
Negative cultures	25 (28)

Gram-positive organisms were isolated most often (54/88, 61%), with MRSA and MRSE being the single most common pathogens and Staphylococci in general isolated during 42% of surgeries. Vancomycin MICs were noted as 1 μg/nL in six gram-positive isolates and 2 μg/mL in nine. Other perioperative cultures were positive around 22 surgeries for additional organisms. Joint cultures were most common (17 surgeries), with positive cultures also obtained from blood (4), urine (1), and a nasal swab (1). MRSA, MSSA, and MRSE were isolated around one, two, and two surgeries, respectively, including one additional MRSE isolate with a vancomycin MIC of 2 μg/mL from a joint culture. Fungi were isolated from eight cultures: Candida spp. (4), C tropicalis (2), C parapsilosis (1), and yeast not otherwise specified (1).

Systemic antimicrobials were used after 80 surgeries, while systemic therapy was not used in eight. Systemic antimicrobial therapy was guided by cultures once available, and agents used are shown in Table 3.

**Table 3:** Antimicrobial use after 88 surgeries ^a^Ampicillin, caspofungin, ciprofloxacin, meropenem, metronidazole, micafungin, penicillin G

Antibiotic	n (%)	Antibiotic	n (%)
Cefazolin	2 (2)	Nafcillin	6 (7)
Cefepime	6 (7)	Piperacillin-tazobactam	2 (2)
Ceftriaxone	7 (8)	Rifampin	15 (17)
Daptomycin	44 (50)	Tigecycline	2 (2)
Doxycycline	3 (3)	Vancomycin	16 (18)
Fluconazole	7 (8)	Other^a^	7 (8)
Levofloxacin	13 (15)		

Agents were changed to the oral route when possible. Systemic daptomycin was used after half of the surgeries, often in combination with rifampin and/or culture-directed agents.

Thirty-six of the 53 episodes of PJI (70%) were classified as cured. There were five failures (9%), consisting of four patients who died with PJI and four who received chronic prophylaxis with oral doxycycline. An additional eight patients were lost to follow up. Patients were assessed as a cure among 65% (13/20) and 70% (23/33) of patients with PJI of the hip and knee, respectively. Two patients with artificial hips and two with artificial knees died, and four patients with knee PJI received chronic prophylaxis and was classified as a failure; the remainder were lost to follow up. The cure rate was 92% (11/12) for patients who had any infection with methicillin-resistant staphylococci during the evaluation period, compared with 62% (13/21) for patients with methicillin-susceptible staphylococci. Four MRSA patients received chronic prophylaxis and were classified as a failure; the others were all lost to or currently in follow up. Among patients who had staphylococci with a vancomycin MIC of 1 μg/mL and 2 μg/mL, the cure rates were 71% (5/7) and 44% (4/9), respectively, compared with 85% (17/20) for patients with *Staphylococci* with lower MICs. 

**Figure 1:**
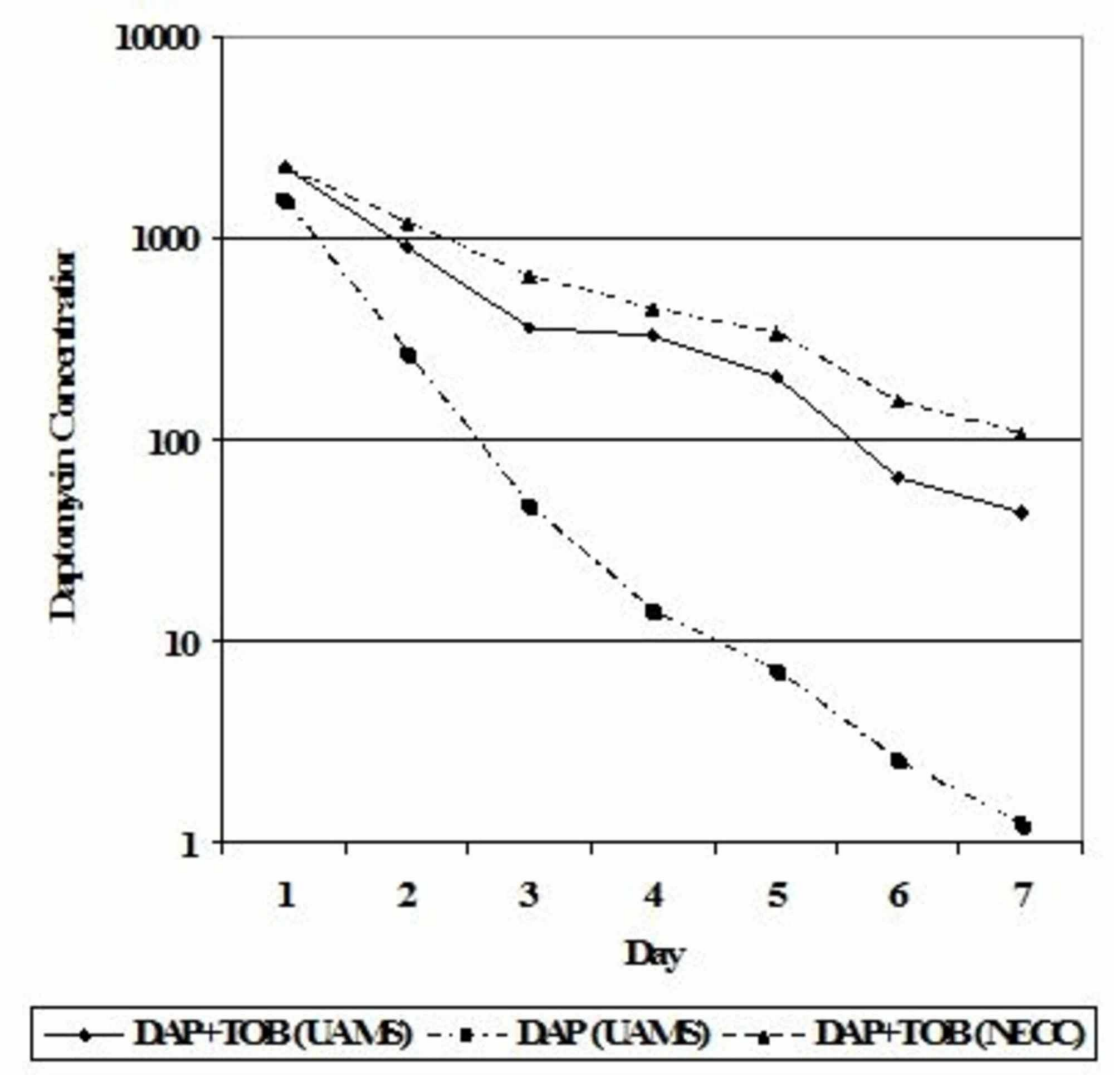
Daptomycin elution from PMMA beads, with and without tobramycin, from beads prepared at our center or prefabricated at the New England Compounding Center DAP, daptomycin; NECC, New England Compounding Center; TOB, tobramycin; UAMS, University of Arkansas for Medical Sciences; PMMA, polymethylmethacrylate

The addition of tobramycin significantly enhanced the elution of daptomycin over the first week (Figure 1), both for beads made onsite and those obtained commercially. On days one and seven, the addition of tobramycin increased daptomycin concentrations by 47% and 3374%, respectively, for beads made onsite compared to elution of daptomycin alone.

Elution increased by 44% and 8394%, respectively, for commercial beads compared to beads fabricated onsite. For beads made onsite or obtained commercially, daptomycin concentrations were well above MIC breakpoints on day seven.

Intra-articular daptomycin levels were measured in six patients and were ranging from 25-886 μg/mL on days two and 30 after surgery. One patient with chronic renal failure had joint levels that were much lower than the others. Serum daptomycin concentrations were also obtained in these patients and ranged from 12-49 μg/mL on days two and 30, respectively. Serum levels of daptomycin were consistent between the patients. In both serum and joint, measured daptomycin concentrations remained over the susceptibility breakpoint MIC for all organisms.

## Discussion

A two-stage arthroplasty with the use of a temporary antibiotic-impregnated device has become the treatment of choice in the US for most chronic PJI [20-22]. Antibiotic-impregnated beads have been used since 1972 [23]. Local administration of antibiotics can achieve concentrations that are higher than via the systemic route, allowing improved diffusion to avascular areas of the wound and higher concentrations relative to the MIC of the infecting organism with limited systemic adverse events [5]. Incorporated antimicrobials should be available as a powder, stable to the heat generated during polymerization of PMMA, active against organisms typically associated with PJI, and cause minimal adverse events by local delivery [24]. Antibiotic beads have become increasingly popular as resistance rates have risen among pathogens commonly associated with PJI.

Although vancomycin has long been a standard for incorporation into local devices, resistance has been emerging to this antibiotic. As previously mentioned, MIC creep and decreased the efficacy of vancomycin against susceptible organisms with MICs ≥1 μg/mL have been increasingly reported, and hVISA, VISA, and vancomycin-resistant *S. aureus* (VRSA) have been spreading [25]. Among other limitations, systemic vancomycin also requires monitoring of serum concentrations for most patients and has been associated with nephrotoxicity and ototoxicity, particularly with similar toxins or at elevated blood levels, which may be used in the treatment of organisms with elevated MICs [9,11]. In the face of failure with vancomycin, there are currently no defined guidelines for the management of PJI, although the common practice is to treat these infections with a combination of systemic and local vancomycin and often amputation. Systemic daptomycin previously has shown equivalence to vancomycin in an animal model of foreign-body infections [6]. IV daptomycin has been associated with elevations of creatine phosphokinase, but local daptomycin should result in a significantly lower incidence of systemic adverse events (although creatine phosphokinase levels were not collected in this study) [26-27]. Although some vancomycin-nonsusceptible isolates, particularly hVISA and VISA, have also been nonsusceptible to daptomycin, daptomycin nonsusceptibility remains low, and the clinical significance is unclear when local administration is used [17-18].

Daptomycin has demonstrated favorable in vitro elution characteristics when incorporated as the sole antibiotic into bone cement [24,27-28]. Using a disc diffusion method, daptomycin 2.5% included in various dextran-containing PMMA formulations exhibited good release characteristics while maintaining its antibiotic efficacy [28]. In a continuous flow chamber, daptomycin 2.5%, 7.5%, and 15% were shown to elute from 3-mm PMMA beads at rates similar to previously studied vancomycin 7.5% [24]. Increasing the concentration increased the amount of drug eluted in a nonlinear fashion, with daptomycin 7.5% and 15% exceeding vancomycin 7.5%; a daptomycin concentration of 8.2% was used in this series. Besides, protein binding, which is high for daptomycin, is not addressed by this method [24,29]. As expected, this report showed daptomycin elution to be enhanced by the addition of tobramycin, likely because the aminoglycoside acted as a “filler,” albeit an active one, increasing the porosity of the bone cement. Beads containing daptomycin previously have been shown to be effective in humans with vascular graft infections, although clinical outcomes have not been reported before for PJI [30]. In vitro activity and release characteristics also have been demonstrated to be favorable for linezolid, but release characteristics have not been evaluated for other new agents that are alternatives to vancomycin; clinical outcomes have not been reported for any of these drugs [17].

Intra-articular concentrations of daptomycin were 25-886 μg/mL, compared to a steady-state maximum serum concentration (Cmax) of 94 μg/mL and a minimum concentration (Cmin) of 7 μg/mL after administration of 6 mg/kg IV to healthy volunteers over 30 min as per study conducted by Cubist Pharmaceuticals in 2010. Intra-articular levels exceeded the reported Cmax at all times in the first patient, while levels in the second patient surpassed the known Cmax part of the time and the Cmin at all times. Joint levels of daptomycin varied substantially between patients, suggesting that a number of factors may affect intra-articular concentrations. In this study, concentrations in the joint remained in excess of the MIC90 for MRSA and VRE for less than or equal to five weeks postoperatively. Thus, daptomycin-PMMA beads would be predicted to be effective against the majority of *Staphylococci* and *Enterococci*. Systemic daptomycin concentrations were not shown to be elevated in this study due to the implantation of daptomycin-containing PMMA beads, with random serum concentrations of 12‑49 μg/mL. Therefore, IV daptomycin does not seem to require a dose adjustment when daptomycin-PMMA beads are used in combination.

Device-associated infections are usually caused by biofilm-producing organisms; biofilm-producing organisms are relatively resistant to therapeutic concentrations of most antimicrobials, regardless of any underlying resistance [1,5]. Because antimicrobials achieve much higher concentrations when administered locally than systemically, they may overcome this relative resistance [5]. Daptomycin maintains activity against established biofilms [6]. Daptomycin has shown efficacy similar to or greater than vancomycin, linezolid, or tigecycline against biofilm-producing staphylococci [6]. Unlike the cell wall-active vancomycin or the protein synthesis inhibitor linezolid, daptomycin, which works by inserting itself into the bacterial cell membrane, maintains bactericidal activity against slowly growing or nongrowing bacteria primarily due to the activity of daptomycin against biofilm-producing organisms, this agent has replaced vancomycin for local use in our practice [17].

Patients in this study were evaluated retrospectively and thus are subject to the usual limitations of a retrospective trial, particularly the impossibility of controlling for comorbidities and other known risk factors for PJI. Elution of tobramycin from PMMA has previously been shown to be comparable to gentamicin [28]. Because of the high concentrations achieved, locally administered aminoglycosides may have activity against *Staphylococci*; thus, an effect of the tobramycin could not be excluded. However, decreased susceptibility to gentamicin and tobramycin has been reported for *Staphylococci *associated with PJI in the US [1].

Among other variables, antibiotic elution from PMMA is dependent on the surface area of the bead, the absolute amount of drug contained, the presence of more than one antimicrobial, the specific vehicle used, and the presence of fillers [1]. Therefore, beads that differ from these significantly may not achieve the same results. Also, the correct rate of in vivo extracellular fluid replacement in infected joints is not known. This may differ among patients and may not reflect the constant flow rate used in in vitro experiments, including our elution evaluation. Finally, PMMA beads were not supplemented locally with calcium. Free calcium ions are necessary for the bactericidal activity of daptomycin, with a concentration of approximately 50 μg/mL needed for activation of daptomycin, a free calcium concentration similar to that seen in the blood of healthy adults [28]. However, calcium levels may vary in locations of compromised vascular status, such as infected joints [6]. Daptomycin-impregnated beads were effective in our patients, but local calcium levels may be considered in patients in whom daptomycin does not work.

## Conclusions

Daptomycin with tobramycin-loaded PMMA beads can be a safe and powerful bactericidal local antibiotic delivery system in biofilm-producing bacteria especially in recurrent or resistant prosthetic joint infections due to the significantly high local drug levels delivered in fatal concentrations for the bacteria.

## References

[1] Bedair H, Goyal N, Dietz MJ (2015). A history of treated periprosthetic joint infection increases the risk of subsequent different site infection. Clin Orthop Relat Res.

[2] Della Valle C, Parvizi J, Bauer TW (2011). American Academy of Orthopaedic Surgeons clinical practice guideline on: the diagnosis of periprosthetic joint infections of the hip and knee. J Bone Joint Surg Am.

[3] Belden K, Cao L, Chen J (2019). Hip and knee section, fungal periprosthetic joint infection, diagnosis and treatment: Proceedings of International Consensus on Orthopedic Infections. J Arthroplasty.

[4] Del Pozo JL, Patel R (2009). Clinical practice. Infection associated with prosthetic joints. N Engl J Med 361.

[5] Diefenbeck M, Muckley T, Hofmann GO (2006). Prophylaxis and treatment of implant-related infections by local application of antibiotics. Injury.

[6] Rice DA, Mendez-Vigo L (2009). Daptomycin in bone and joint infections: a review of the literature. Arch Orthop Trauma Surg.

[7] Leibovici L, Vidal L, Paul M (2009). Aminoglycoside drugs in clinical practice: an evidence-based approach. J Antimicrob Chemother.

[8] Bayer AS, Murray BE (2009). Initial low-dose aminoglycosides in Staphylococcus aureus bacteremia: good science, urban legend, or just plain toxic?. Clin Infect Dis.

[9] Rybak MJ, Lomaestro BM, Rotschafer JC (2009). Therapeutic monitoring of vancomycin in adults summary of consensus recommendations from the American Society of Health-System Pharmacists, the Infectious Diseases Society of America, and the Society of Infectious Diseases Pharmacists. Pharmacotherapy.

[10] Masri BA, Duncan CP, Beauchamp CP ( 1998). Long-term elution of antibiotics from bone-cement: an in vivo study using the prosthesis of antibiotic-loaded acrylic cement (PROSTALAC) system. J Arthroplasty.

[11] Kollef MH (2007). Limitations of vancomycin in the management of resistant staphylococcal infections. Clin Infect Dis.

[12] Cacciola G, De Meo F, Cavaliere P (2018). Mechanical and elution properties of G3 Low Viscosity bone cement loaded up to three antibiotics. J Orthop.

[13] Hidayat LK, Hsu DI, Quist R, Shriner KA, Wong-Beringer A (2006). High-dose vancomycin therapy for methicillin-resistant Staphylococcus aureus infections: efficacy and toxicity. Arch Intern Med.

[14] Haddad FS, Masri BA, Campbell D, McGraw RW, Beauchamp CP, Duncan CP (2000). The PROSTALAC functional spacer in two-stage revision for infected knee replacements. Prosthesis of antibiotic-loaded acrylic cement. J Bone Joint Surg Br.

[15] Castanheira M, Jones RN, Sader HS ( 2008). Update of the in vitro activity of daptomycin tested against 6710 Gram-positive cocci isolated in North America (2006). Diagn Microbiol Infect Dis.

[16] Wang G, Hindler JF, Ward KW, Bruckner DA (2006). Increased vancomycin MICs for Staphylococcus aureus clinical isolates from a university hospital during a 5-year period. J Clin Microbiol.

[17] Sader HS, Becker HK, Moet GJ, Jones RN (2010). Antimicrobial activity of daptomycin tested against Staphylococcus aureus with vancomycin MIC of 2 microg/mL isolated in the United States and European hospitals (2006-2008). Diagn Microbiol Infect Dis 66.

[18] Moise PA, North D, Steenbergen JN, Sakoulas G (2009). Susceptibility relationship between vancomycin and daptomycin in Staphylococcus aureus: facts and assumptions. Lancet Infect Dis.

[19] Sader HS, Moet G, Jones RN (2009). Update on the in vitro activity of daptomycin tested against 17,193 Gram-positive bacteria isolated from European medical centers (2005-2007). J Chemother.

[20] Gooding CR, Masri BA, Duncan CP, Greidanus NV, Garbuz DS (2011). Durable infection control and function with the PROSTALAC spacer in two-stage revision for infected knee arthroplasty. Clin Orthop Relat Res.

[21] Biring GS, Kostamo T, Garbuz DS, Masri BA, Duncan CP (2009). Two-stage revision arthroplasty of the hip for infection using an interim articulated Prostalac hip spacer: a 10- to 15-year follow-up study. J Bone Joint Surg Br 91.

[22] Citak M, Argenson JN, Masri B (2014). Spacers. J Orthop Res.

[23] Swearingen MC, Granger JF, Sullivan A, Stoodley P (2016). Elution of antibiotics from poly(methyl methacrylate) bone cement after extended implantation does not necessarily clear the infection despite susceptibility of the clinical isolates. Pathog Dis.

[24] Hall EW, Rouse MS, Jacofsky DJ, Osmon DR, Hanssen AD, Steckelberg JM, Patel R (2004). Release of daptomycin from polymethylmethacrylate beads in a continuous flow chamber. Diagn Microbiol Infect Dis.

[25] Howden BP, Davies JK, Johnson PD, Stinear TP, Grayson ML (2010). Reduced vancomycin susceptibility in Staphylococcus aureus, including vancomycin-intermediate and heterogeneous vancomycin-intermediate strains: resistance mechanisms, laboratory detection, and clinical implications. Clin Microbiol Rev.

[26] Fowler VG Jr., Boucher HW, Corey GR (2006). Daptomycin versus standard therapy for bacteremia and endocarditis caused by Staphylococcus aureus. N Engl J Med.

[27] Webb ND, McCanless JD, Courtney HS, Bumgardner JD, Haggard WO (2008). Daptomycin eluted from calcium sulfate appears effective against Staphylococcus. Clin Orthop Relat Res.

[28] Kuechle DK, Landon GC, Musher DM, Noble PC (1991). Elution of vancomycin, daptomycin, and amikacin from acrylic bone cement. Clin Orthop Relat Res.

[29] Dvorchik B, Damphousse D (2004). Single-dose pharmacokinetics of daptomycin in young and geriatric volunteers. J Clin Pharmacol.

[30] Stone PA, Armstrong PA, Bandyk DF (2006). Use of antibiotic-loaded polymethylmethacrylate beads for the treatment of extracavitary prosthetic vascular graft infections. J Vasc Surg.

